# Enhancing electric vehicle charging performance through series-series topology resonance-coupled wireless power transfer

**DOI:** 10.1371/journal.pone.0300550

**Published:** 2024-03-21

**Authors:** Nadir Benalia, Idriss Benlaloui, Kouider Laroussi, Ahmad Elkhateb, Daniel Eutyche Mbadjoun Wapet, Ammar M. Hassan, Mohamed Metwally Mahmoud

**Affiliations:** 1 Faculty of Science and Technology, Department of Electrical Engineering, Laboratory of Applied Automation and Industrial Diagnostics (LAADI), Ziane Achour University, Djelfa, Algeria; 2 Department of Electrical Engineering, Laboratory LSPIE, University of Batna, Batna, Algeria; 3 School of Electronics, Electrical Engineering and Computer Science (EEECS), Queen’s University, Belfast, United Kingdom; 4 National Advanced School of Engineering, Universit´e de Yaound´e I, Yaound´e, Cameroon; 5 Arab Academy for Science, Technology and Maritime Transport, South Valley Branch, Aswan, Egypt; 6 Faculty of Energy Engineering, Department of Electrical Engineering, Aswan University, Aswan, Egypt; Pandit Deendayal Petroleum University, India, INDIA

## Abstract

The current electric vehicles (EVs) market is experiencing significant expansion, underscoring the need to address challenges associated with the limited driving range of EVs. A primary focus in this context is the improvement of the wireless charging process. To contribute to this research area, this study introduces a circular spiral coil design that incorporates transceiver coils. First, an in-depth analysis is conducted using Ansys Maxwell software to assess the effectiveness of the proposed solution through the magnetic field distribution, inductance properties, and mutual inductance between receiver and transmitter coils. In the next step, a direct shielding technique is applied, integrating a ferrite core bar to reduce power leakage and enhance power transmission efficiency. The ferrite magnetic shielding guides magnetic field lines, resulting in a significant reduction in flux leakage and improved power transmission. Lastly, a magnetic resonance series (SS) compensation wireless system is developed to achieve high coupling efficiency and superior performance. The system’s effectiveness is evaluated through co-simulation using Ansys Simplorer software. The results confirm the effectiveness of the proposed solution, showing its ability to transmit 3.6 kilowatts with a success rate approaching 99%. This contribution significantly advances the development of wireless charging systems for electric vehicles, addressing concerns and promoting global adoption.

## 1. Introduction

The contemporary global shift towards electric vehicles (EVs) is driven by a dual motivation: the imperative to reduce polluting emissions from traditional fossil fuel-powered cars, and the economic advantages associated with lower maintenance costs and the use of cleaner energy alternatives [1–3]. However, despite these advantages, the widespread adoption of EVs is hindered by two limiting factors: driving range and the charging process [4–6].

In response to these challenges, researchers are exploring wireless power (WP) Transmission as a superior alternative to conventional wired connections [7,8]. Wireless charging technology utilizes magnetic fields to induce the transfer of electrical current between remote coils, presenting an effective solution to the recharging conundrum and eliminating the need for physical connections or cables. Thanks to the pioneering work of Nikola Tesla, inductive resonant coupling, a wireless charging method employing magnetic resonance, facilitates efficient and secure power transfer across short distances [9,10].

This technology involves a charging pad or coil placed on the ground or within a parking spot. When an EV parks over the pad, a magnetic field is generated, inducing an alternating current in a receiving coil on the vehicle’s underside. This induced electricity charges the vehicle’s batteries, providing a reliable and convenient charging method [11,12]. Charging methods include static charging (when the vehicle is stationary), stationary charging (at designated areas, even while the vehicle’s engine is operational), and dynamic charging (while the vehicle is moving on a road equipped with electronic infrastructure). Circular coil configurations are commonly employed in WP transfer systems for charging EV batteries, with various discussions and enhancements documented in references [13]. Notably, [14], and [15] emphasize the prevalence and improvements in circular coil designs. In [12], the file system is configured as a plate, containing file elements, flow path materials, and protective materials. The use of MnZn ferrite materials helps restrict flow to the intended path and reduce leakage in the surrounding area [16]. The efficiency of the system is further enhanced by utilizing ferrite plates or bars with an insulating substance between them and the coil conductors, as proposed in multiple research works [17–19].

Despite advancements, the current state of WP transfer for EVs faces a significant challenge: the decoupling of board design and power electronics design. A holistic approach recognizing the interplay between primary coil parameters and overall structure effectiveness is crucial for advancing efficient and integrated WPT solutions [20,21]. Several global demonstrations and pilot projects involving dynamic wireless power transfer (DWPT) have been undertaken by researchers, vehicle manufacturers, and energy companies. Notable examples include the UNPLUGGED and FABRIC European project, focusing on the effects of EV driving range [22–24]. Reference [25] introduced a novel methodology and control system for bidirectional WP Transfer systems, emphasizing the cascade series compensation topology. It is important to note the primary focus on coil design, neglecting the interrelation between plate design and targeted energy transfer. In [26,27], the authors aim to improve the electrical properties of inductive wireless systems, emphasizing the correlation between charging coil geometric characteristics and overall system efficiency affecting energy transfer.

Acknowledging previous research initiatives in [28] that explore the intricacies of designing and optimizing WPT systems, it is noteworthy that these studies did not delve into the integration of ferrite materials within the design process [28,29]. This paper presents an in-depth analysis of WP transfer employing a series-series (S-S) topology resonant system featuring circular spiral-shaped coils specifically designed for electric vehicles. The research yields the following key contributions:

•The investigation of circular spiral coil structures and the corresponding mathematical analysis crucial for calculating their parameters, such as Din, Dout, wire length, inductance determination, and total resistance at a frequency of 85 kHz. This involves defining the number of turns required to achieve the desired inductance for efficient power transfer.

•Highlighting the insufficient coupling coefficient and significant leakage flux observed in the obtained results. To mitigate these issues and enhance the power transfer system’s performance, the introduction of ferrite bars for shielding purposes is proposed. Additionally, the analysis of self and mutual induction parameters, along with a 3D assessment of magnetic field distribution using Ansys Maxwell software, is conducted.

• Emphasizing the necessity of establishing a strong magnetic field to efficiently link receiving coils can be accomplished by providing adequate current or magneto-motive force. To diminish the apparent power rating of the power supply, a compensation method is suggested, which entails incorporating a capacitor in series with the transmitting coil. This compensation technique not only reduces apparent power consumption but also improves the system’s ability to adapt to shifts in the factor of coupling within the WP transfer system. The S-S topology is advocated for its independence from coupling coefficient and load variations [29], ensuring consistent resonance frequency, even under misalignment. Additionally, the benefits of this topology, including secondary-side current dependency on the AC source rather than the induced voltage at the secondary coil, advantageous for battery charging, are highlighted. The development of a magnetic resonance series compensation wireless system using a co-simulation approach through Ansys Simplorer is validated through simulation results, demonstrating an impressive capacity to transmit up to 3.6 kilowatts of energy with a success rate nearing 99%.

•Utilizing MATLAB, the paper provides a detailed analysis of parameters such as self-inductance, resistance, currents, power, and coupling coefficient based on the provided system diagram and descriptions. These calculations are pivotal in comprehending and optimizing the wireless power transfer system for an output of approximately 3.6 kW. The optimization process involves adjustments in parameters like coil dimensions, wire characteristics, turn count, and coil distances to enhance efficiency. Ensuring resonance between coils at the designated frequency, evaluating losses, employing high-conductivity materials, minimizing skin effect, and optimizing mutual inductance are recommended strategies for achieving high-power wireless energy transfer.

Continuous refinement and optimization of these parameters, guided by simulations, experiments, and further analysis, are proposed avenues to achieve the desired high-power wireless energy transfer efficiently.

The document is structured as follows: Section 2 delves into the analysis of the series resonance system. Section 3 focuses on the calculation of design parameters for circular spiral coils. Section 4 offers the influence of system efficiency due to the comprehensive S-S circuit; the conclusion is outlined within Section 5.

## 2. Analysis of series resonance compensation

**Fig 1:**
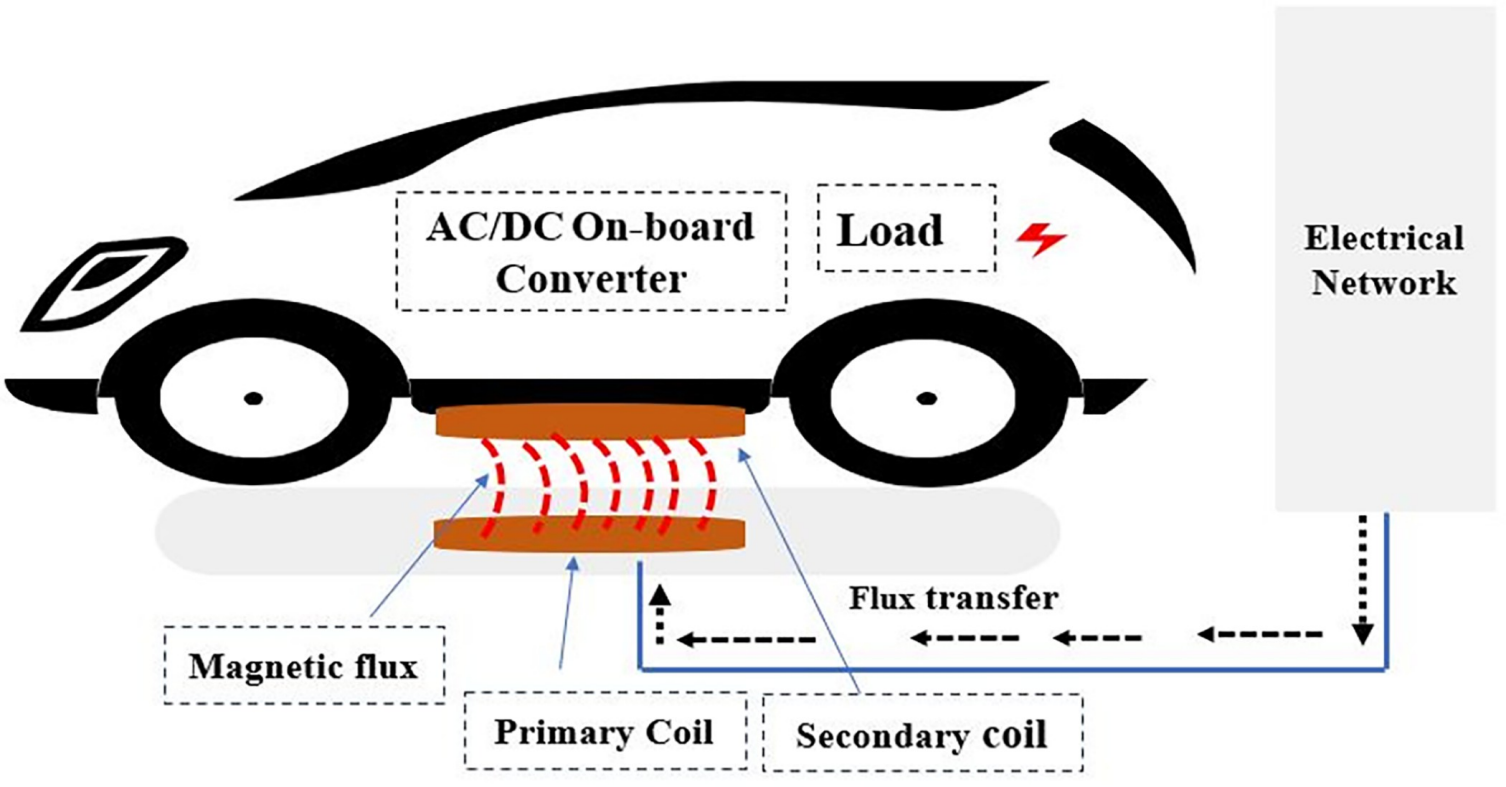
Wireless charging EV.

Wireless charging streamlines vehicle charging by eliminating physical connections and reducing associated time and effort, as illustrated in Fig 1. This method comprises three modes: stationary, dynamic, and resonant.

**Fig 2:**
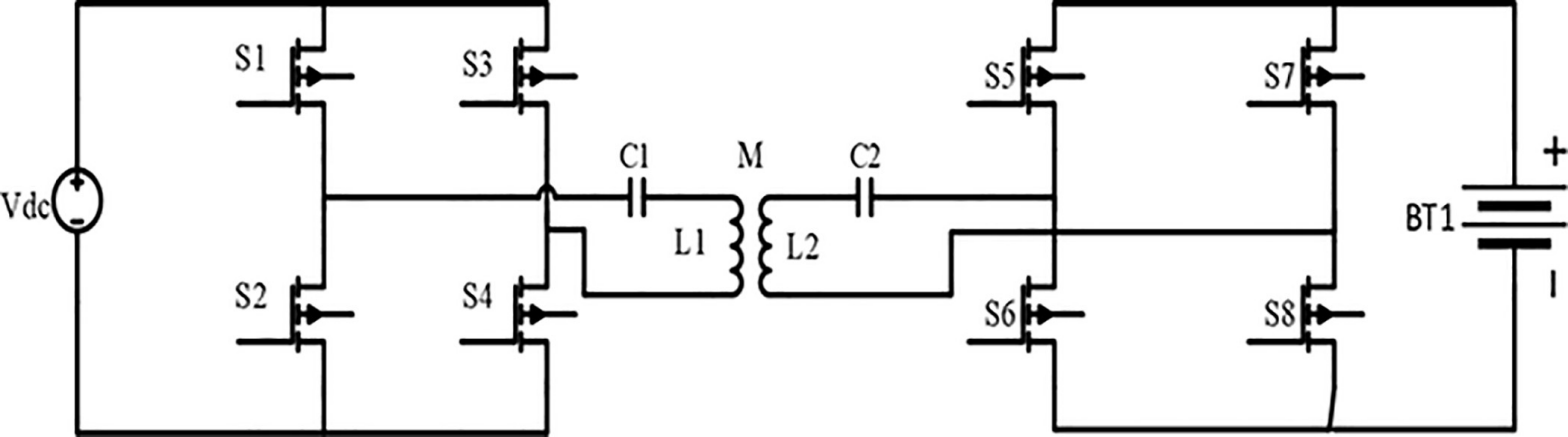
A Comprehensive investigation of S-S WPT for EV charging.

**Fig 3:**
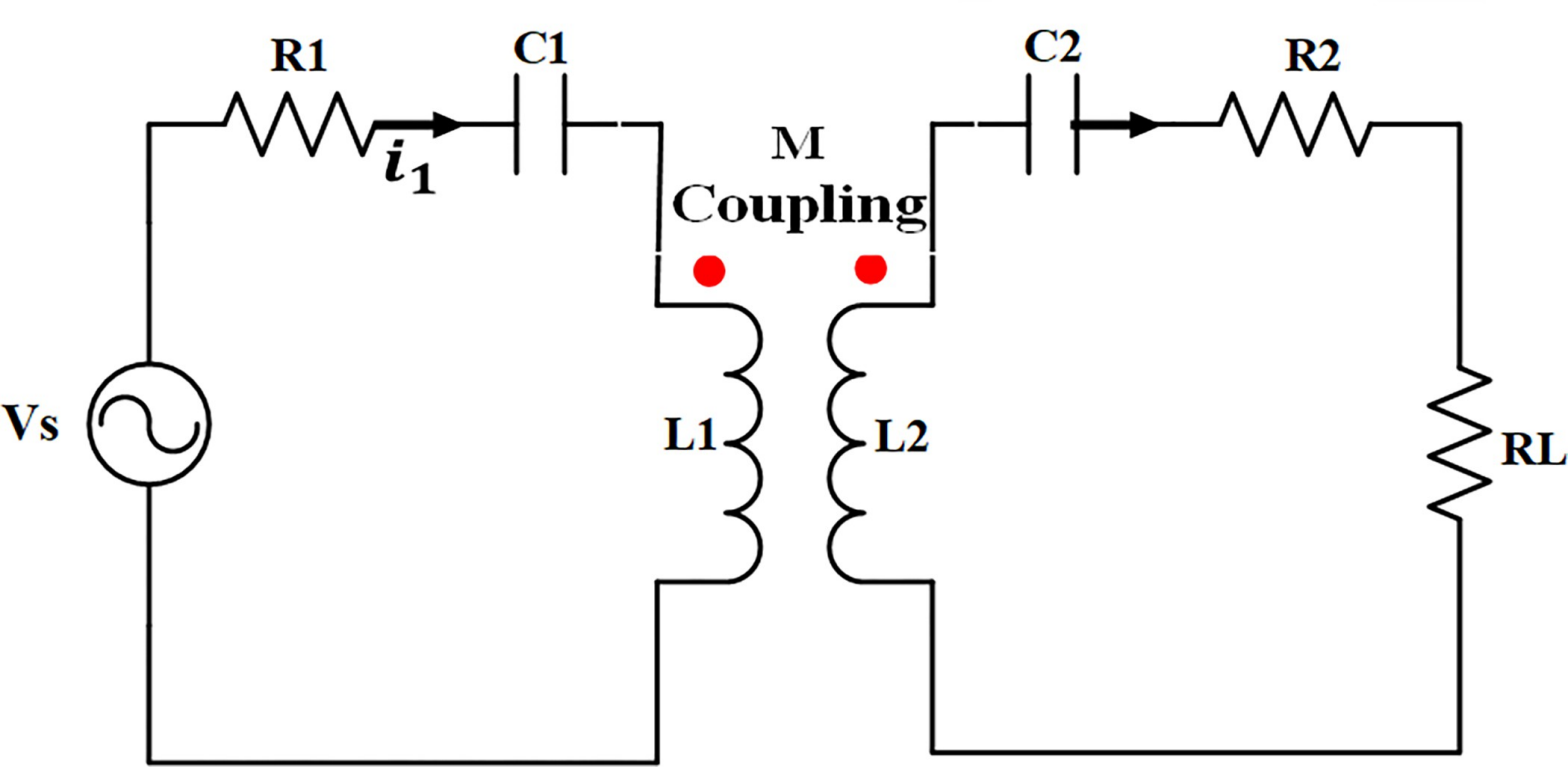
Circuit representation of magnetic resonance WPT.

The selection of the S-S topology is based on its capacity to optimize power transfer, minimize energy losses, and improve charging efficiency [30,31]. This topology employs high-frequency resonant converters for efficient long-distance power transfer, reducing the size and weight of systems for compact EVs. Furthermore, it enables bidirectional power transmission, enabling EVs to supply power to the grid during peak demand and bolstering grid stability. Fig 2 presents a thorough analysis of S-S WPT for EV charging. Fig 3 depicts a schematic representation of the circuit model used for magnetic resonance wireless power transfer.

The resonance inductive coupling WPT system utilizes capacitors and coils during resonance to efficiently transmit power wirelessly [32]. At resonance, inductive reactance (XL) equals capacitive reactance (XC), allowing for maximum current flow, defined by the resonant frequency (ω_0_) determined by the following equation:

$$X_{L}=X_{c}=\omega_{0}L=\frac{1}{\omega_{0}C}\Rightarrow {\omega_{0}}^{2}=\frac{1}{LC}\Rightarrow \omega_{0}=\frac{1}{\sqrt{LC}}$$ (1)

The resonant frequency (*f*_0_) is given by:

$$\omega_{0}=2\pi f_{0}\omega_{0}=\frac{1}{\sqrt{LC}}\Rightarrow f_{0}=\frac{1}{2\pi \sqrt{LC}}$$ (2)

The Eq (3) represents the mathematical model of Fig (3) using Kirchhoff’s voltage law:

$$\left(\begin{array}{c} V_{s}\\ 0 \end{array}\right)=\left(\begin{array}{c} R_{s}+j(\omega L_{1}-\frac{1}{\omega C_{1}})\;\;-j\omega M\\ -j\omega M\;\;R_{L}+j(\omega L_{2}-\frac{1}{\omega C_{2}}) \end{array}\right)\left(\begin{array}{c} I_{s}\\ I_{L} \end{array}\right)$$ (3)

Analyzing (3) mathematically, the expressions for (*I*_*s*_) and (*I*_*L*_) in Fig 3 are reformulated as Eqs (4) and (5), respectively. Furthermore, the efficiency (ɳ) of the WPT system is delineated in Eqs (6) and (7).


$$I_{s}=\frac{V_{s}(R_{L}+R_{1})}{R_{1}(R_{L}+R_{2})+\omega^{2}M^{2}}$$ (4)

$$I_{L}=-\frac{j\omega MV_{s}}{R_{1}(R_{L}+R_{2})+\omega^{2}M^{2}}$$ (5)

$$\eta =\frac{P_{o}}{P_{I}}=\frac{{I_{L}}^{2}R_{L}}{{I_{s}}^{2}Z_{I}}={\left(\frac{I_{L}}{I_{s}}\right)}^{2}*\frac{R_{L}}{Z_{I}}$$ (6)

$$\eta ={\left(\frac{I_{L}}{I_{s}}\right)}^{2}*\frac{\left(R_{L}+R_{1}\right)}{Z_{I}}=\frac{\omega^{2}M^{2}}{R_{1}(R_{L}+R_{2})+\omega^{2}M^{2}}$$ (7)

## 3. Determining key parameters for circular spiral coil design

**Fig 4:**
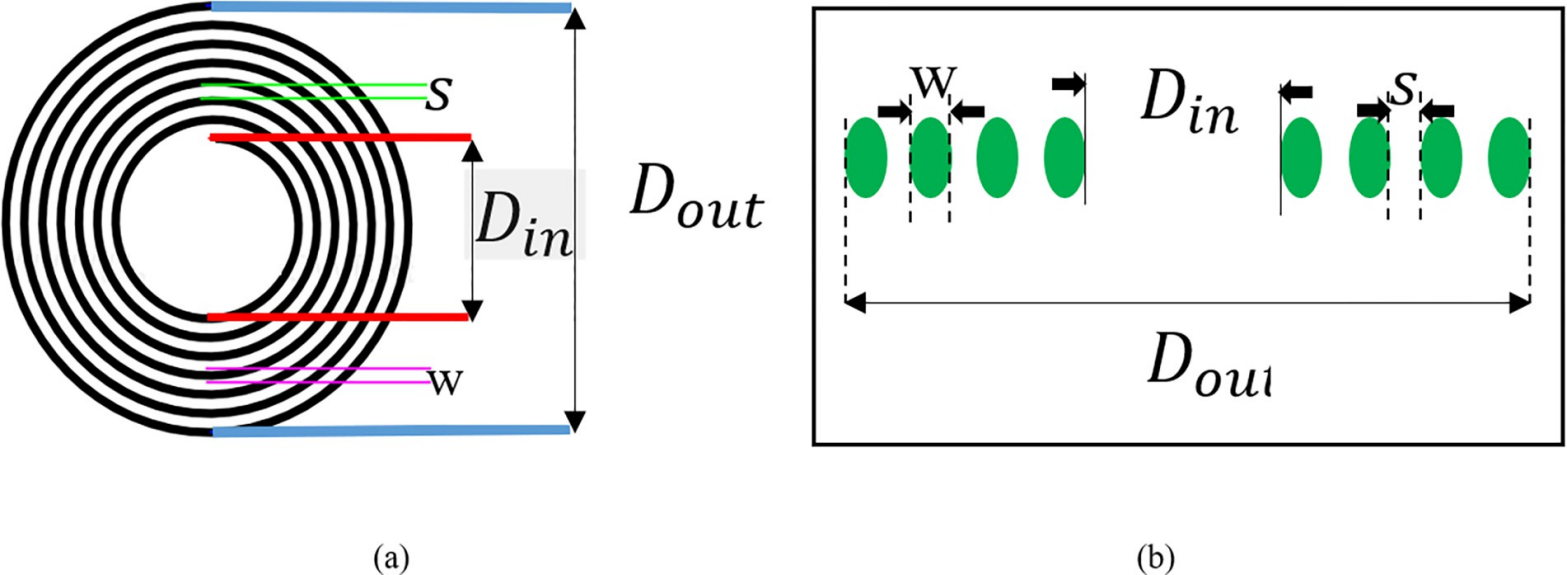
A detailed view of a planar rectangular coil.

To check the special parameters in a circular spiral coil, Fig 4 can be used to analyze the coil. Fig 4 (A) shows the circular spiral coil to be used in this study; Fig 4 (B) shows the given information provides definitions for four key parameters related to a coil [33–35]:

D_out_: This is the outer diameter of the coil.

D_in_: Refers to the inner diameter of the coil.

W: Denotes the diameter of the wire used in the coil.

S: Represents the spacing between the wires of the coil.

Accordingly, L can be calculated by the following equation [36]:

$$L=\frac{N^{2}{\left(D_{out}+D_{in}\right)}^{2}}{20.3(15D_{out}-7D_{in})}$$ (8)

where;

*N*: the number of turns.

To determine both the coil’s outer diameter and the required length, we can employ the following Eqs [9]:

$$D_{out}=D_{in}+(2*N(W+S))$$ (9)

$$W\_length=\frac{\pi N(D_{out}+D_{in})}{2}$$ (10)

### 3.1. Determining of spiral circular coil resistance

The DC resistance of a conductor is contingent upon several factors, including the wire’s length, the cross-sectional area in (m^2^), and the electrical conductivity of the copper material (5.8*10^7^S/m). Additionally, the variable *W*_−*length*_ represents the length of the wire in (m). This situation is seen in (11) [37].

$$Rdc=\frac{W_{-length}}{\sigma .A}$$ (11)

where; *A* = π*r^2^; the cross area in m^2^

*W*_−*length*_: the length of the wire (m)

In the case of taking into account the depth of the skin, the frequency can be added easily by Eq 12, so the total resistance becomes as shown in (13) [38].


$$\delta =\frac{1}{\sqrt{\pi f\sigma \mu }}$$ (12)

$$R_{t}=\frac{l_{w}}{\sigma .A_{w}}\left(\frac{1}{4}+\frac{r}{2\delta }\right)$$ (13)

### 3.2 Case one: Description and analysis of the design of the circular spiral coils

**Fig 5:**
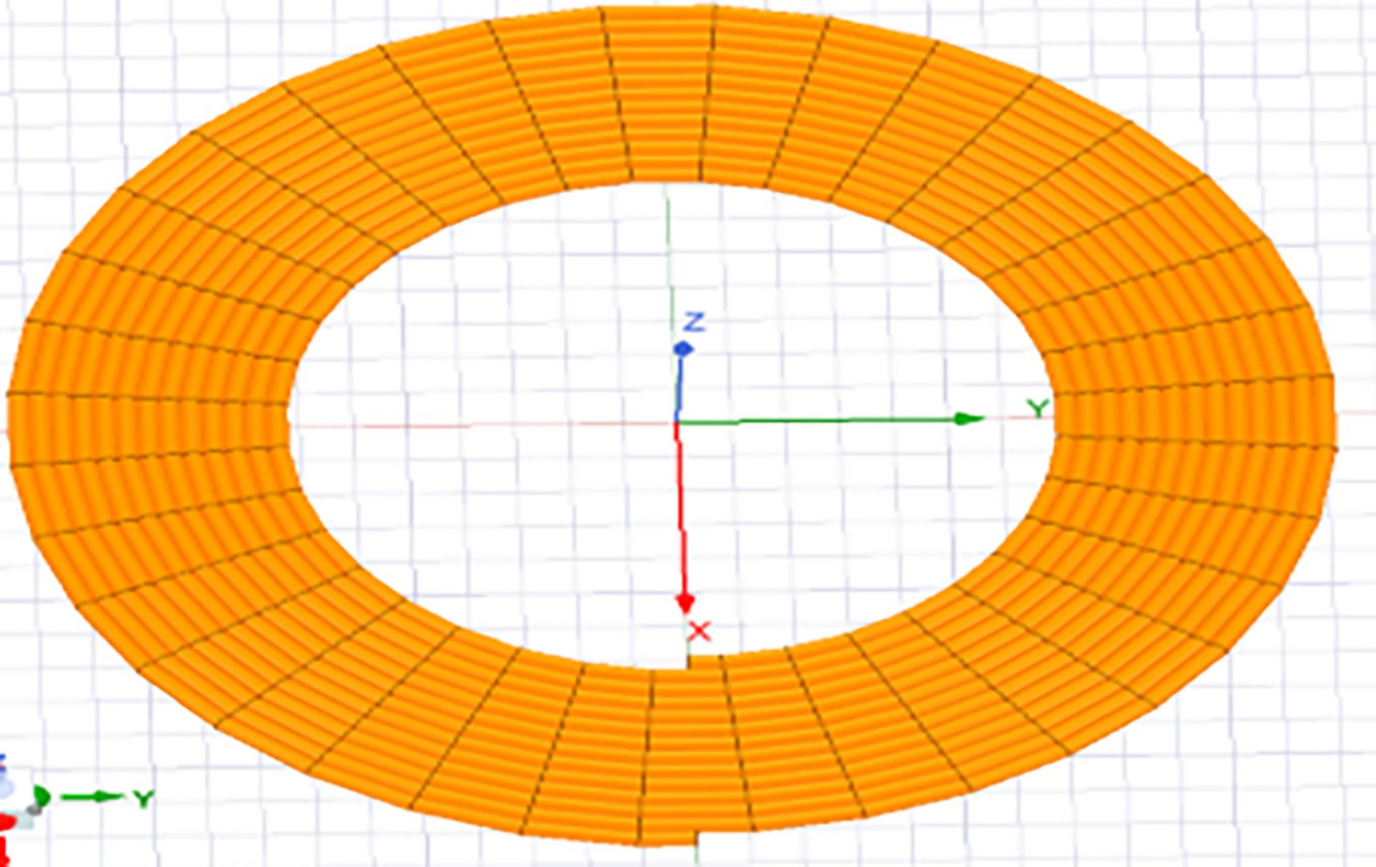
The shape of a circular coil.

In the meticulous design of a spiral circular coil, meticulous consideration must be given to parameters encompassing the inner and outer diameters, the inter-turn spacing, and the total number of turns. These critical design choices are imperative for attaining the desired operational efficiency, as depicted in Fig 5.

**Fig 6:**
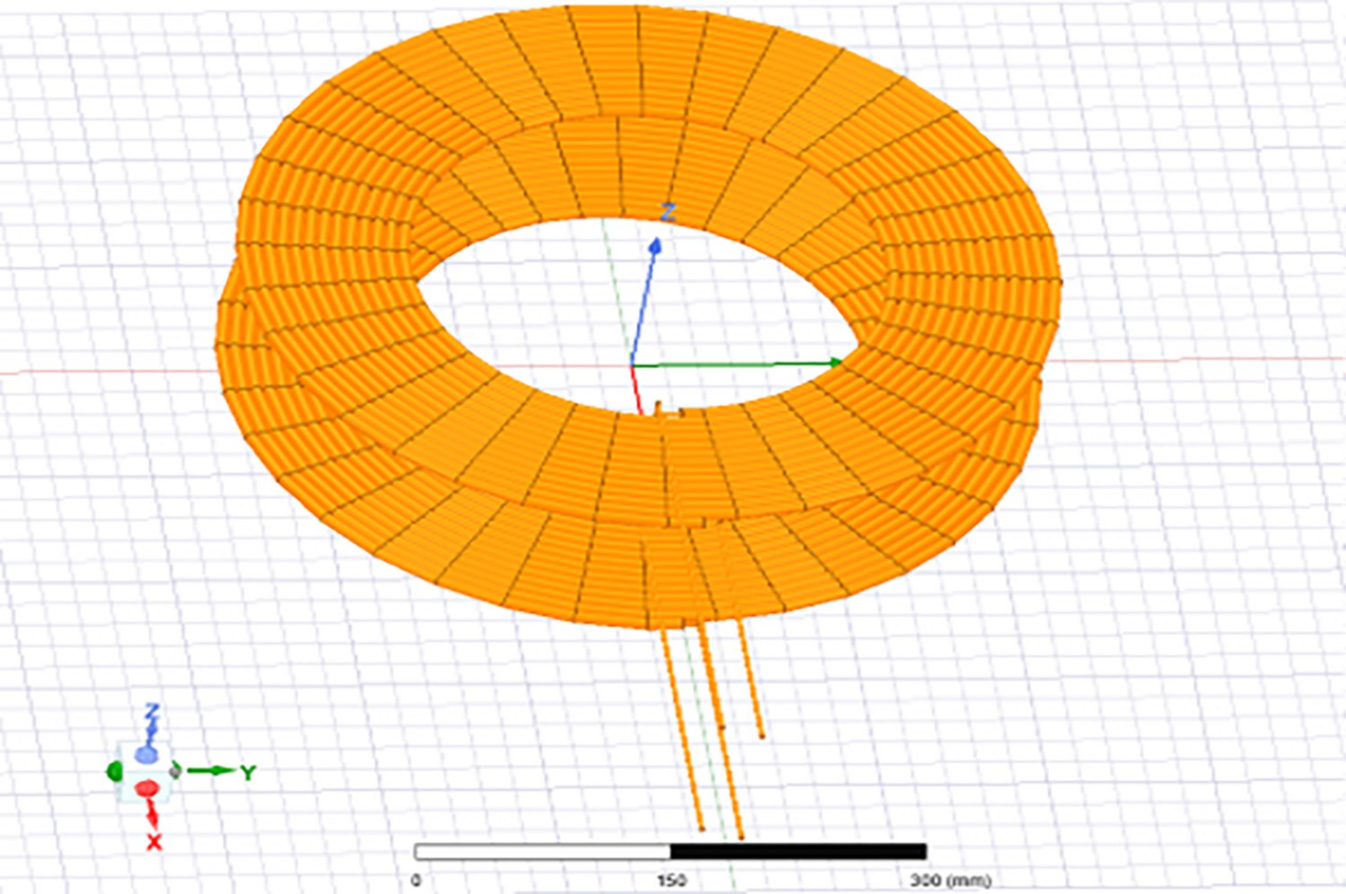
Design of the circular coils with the number of turns N1 = 20, N2 = 20.

Coil 1, referred to as the ground-side coil, and coil 2, known as the vehicle-side coil, have specific dimensions. Their outer diameter measures 495 mm, while the inner diameter is 280 mm. An essential aspect of these coils is that they maintain an air gap of 105 millimeters, which is crucial for ensuring effective coupling between the two coils. This data is graphically represented in Table 1 provides a condensed overview of the geometric parameters derived through the utilization of Eqs (8), (9), and (10)., along with a visual depiction in Fig 6. Furthermore, it has been analyzed through simulation using Ansys Maxwell.

**Table 1:** The parameters of geometry and their calculations summary.

Type	Kind of material	Number of turns	Coil parameter	Pitch	Calculation of parameters
Coil (1)	Copper	13	Dout = 495mm Din = 280mm	0	/Self-Inductance L = 91.846 uH /the length of the wire W_l = 15.84m
Coil (2)	Copper	13	Dout = 495mm Din = 280mm	0	/Self-Inductance L = 91.846 uH /the length of the wire W_l = 15.84m

Ansys Maxwell software is utilized for both the execution and analysis of a transformer that features coils arranged in a spiral configuration. This software contributes an indispensable function in evaluating the structure’s performance. To begin our investigation, we examined the WPT model depicted in Fig 6. Our objective was to gain a thorough understanding of the model and to ascertain key electromagnetic parameters, notably the mutual inductance and coupling coefficient, crucial for energy transfer analysis.

**Fig 7:**
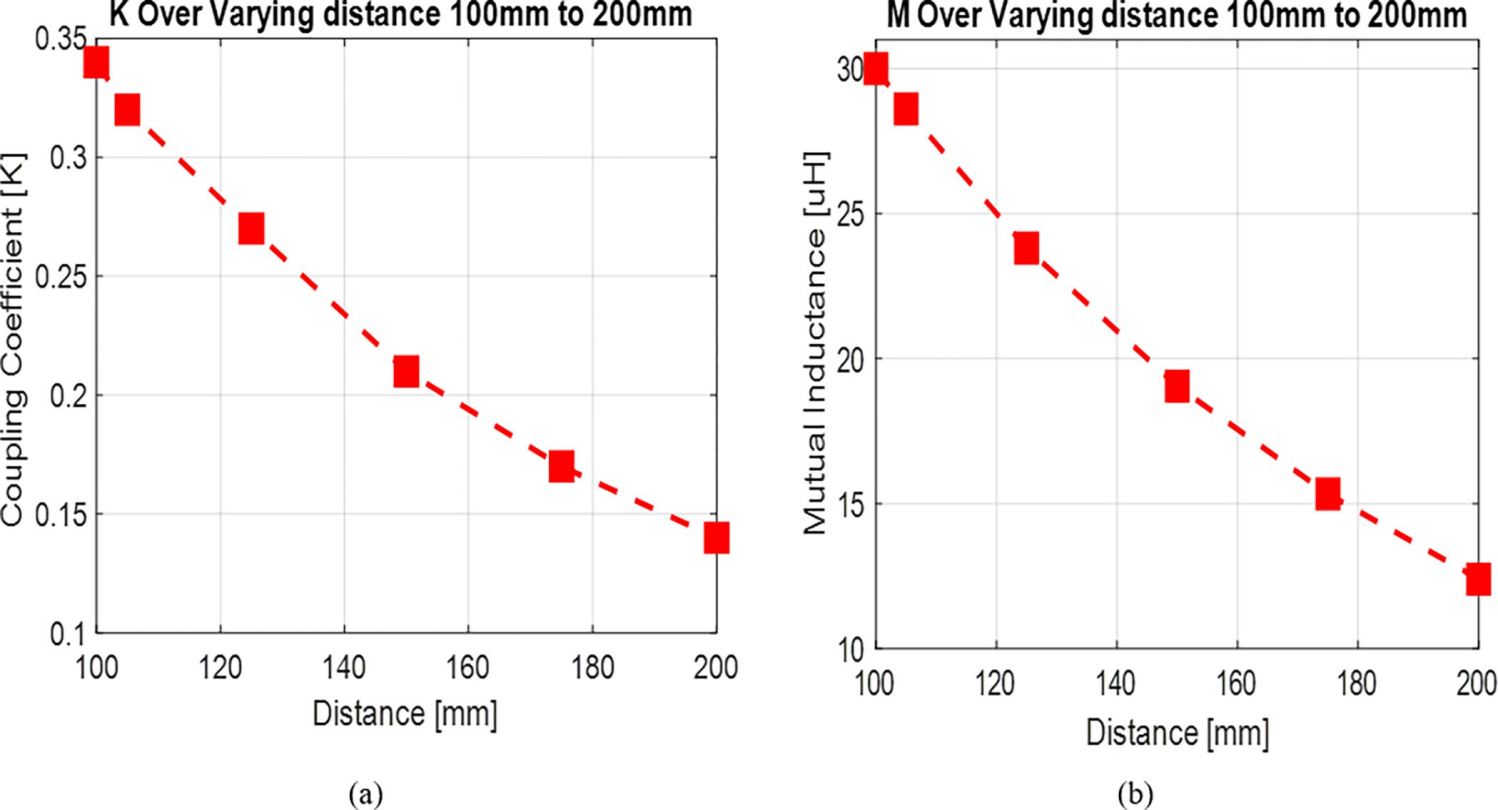
(a) The effect of vertical misalignment (d = 100 mm to 200 mm) on coupling coefficient and (b) mutual inductance.

In Fig 7 we examined the electromagnetic parameters for a system with 13 turns between two coils. As we adjusted the separation distance (d) between these coils within the range of 100 mm to 200 mm, we noted a reduction in both mutual inductance and the coupling coefficient. The decline in these parameters indicates that when the separation distance between two coils expands, the efficiency of energy transfer diminishes. It’s important to note that this relationship is inversely proportional.

**Fig 8:**
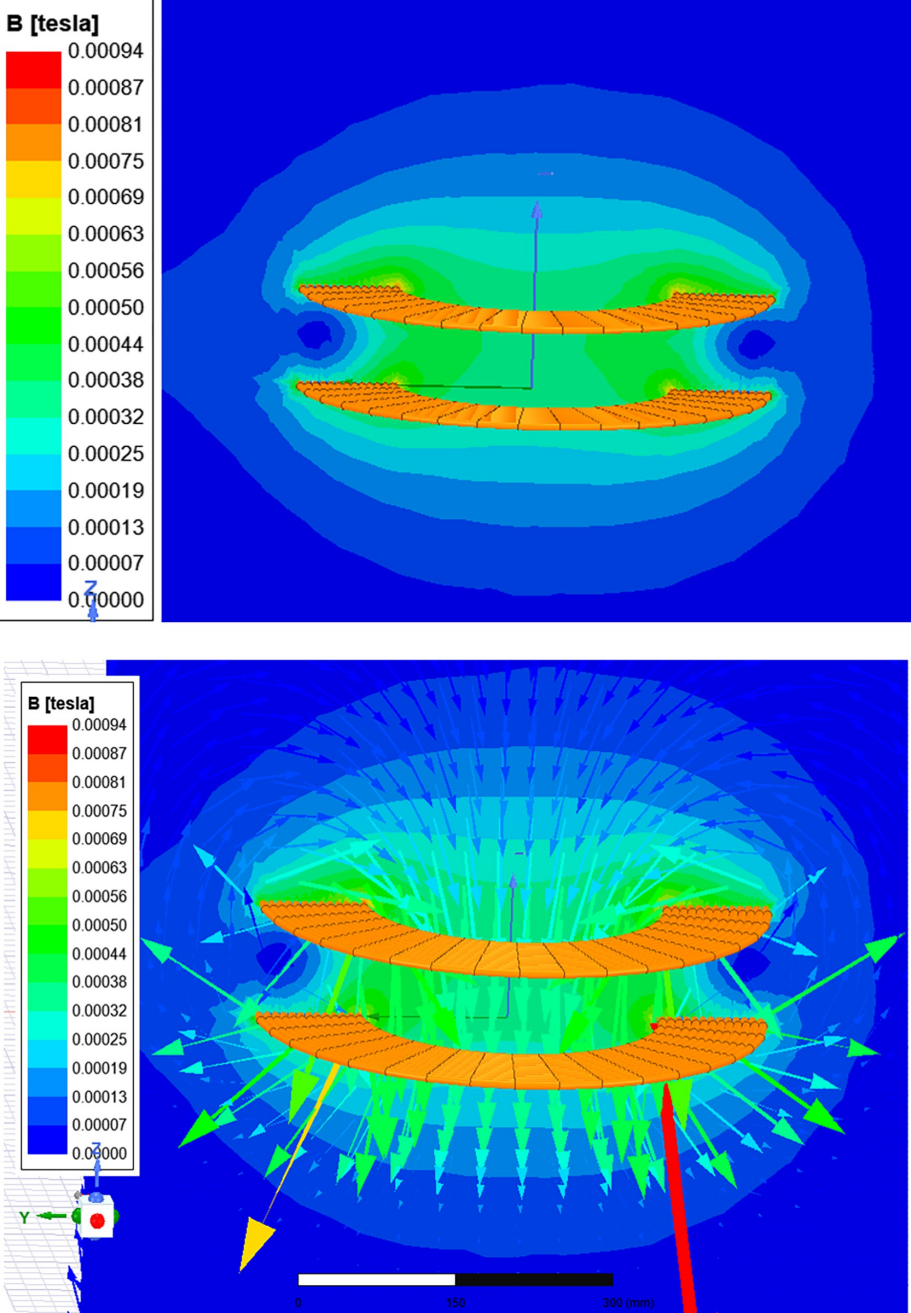
Magnetic field line distribution at a distance of 105 mm.

In Fig 8, the design team applies linear scientific theory to compute electromagnetic fields and flux. This method works well when saturation (a point where magnetic properties change) is not a concern because magnetic induction and flux are directly related to the current in the coil. Permeability refers to a material’s capacity to generate an internal magnetic field when exposed to an external one. Materials with higher permeability align magnetic field lines better, while lower permeability materials like air or a vacuum require shielding to manage electromagnetic effects [5].

### 3.3. Case two: Ferrite on coils (single shielding)

**Fig 9:**
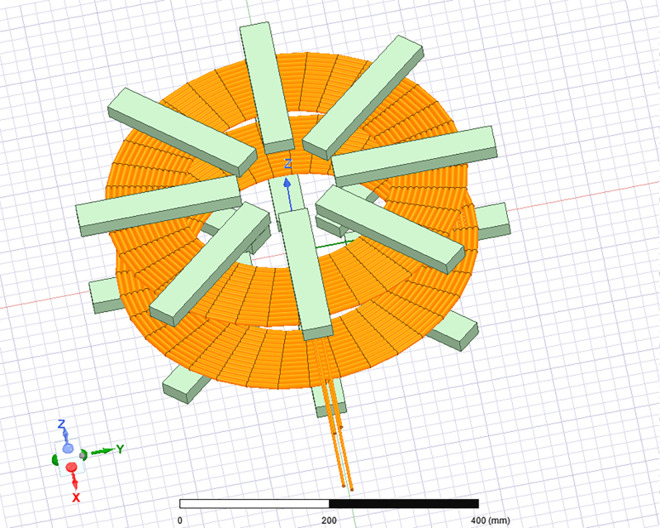
Design of the circular coils with ferrite core bars.

Undoubtedly, in the second phase of this study, the magnetic coupler’s design revolves around establishing the required mutual inductance. It is well-documented that the inclusion of ferrite cores can substantially improve both magnetic shielding and mutual inductance, especially in scenarios featuring a substantial air gap, as illustrated in Fig 9. To kickstart our investigation, we’ve conducted simulations of a magnetic coupler equipped with ferrite cores using Ansoft Maxwell. This process and its outcomes are summarized in Table 2.

**Table 2:** Characteristics of ferrite core in Ansys Maxwell.

Name	Value	Unit
X Size	220	mm
Y Size	40	mm
Z Size	20	mm

**Fig 10:**
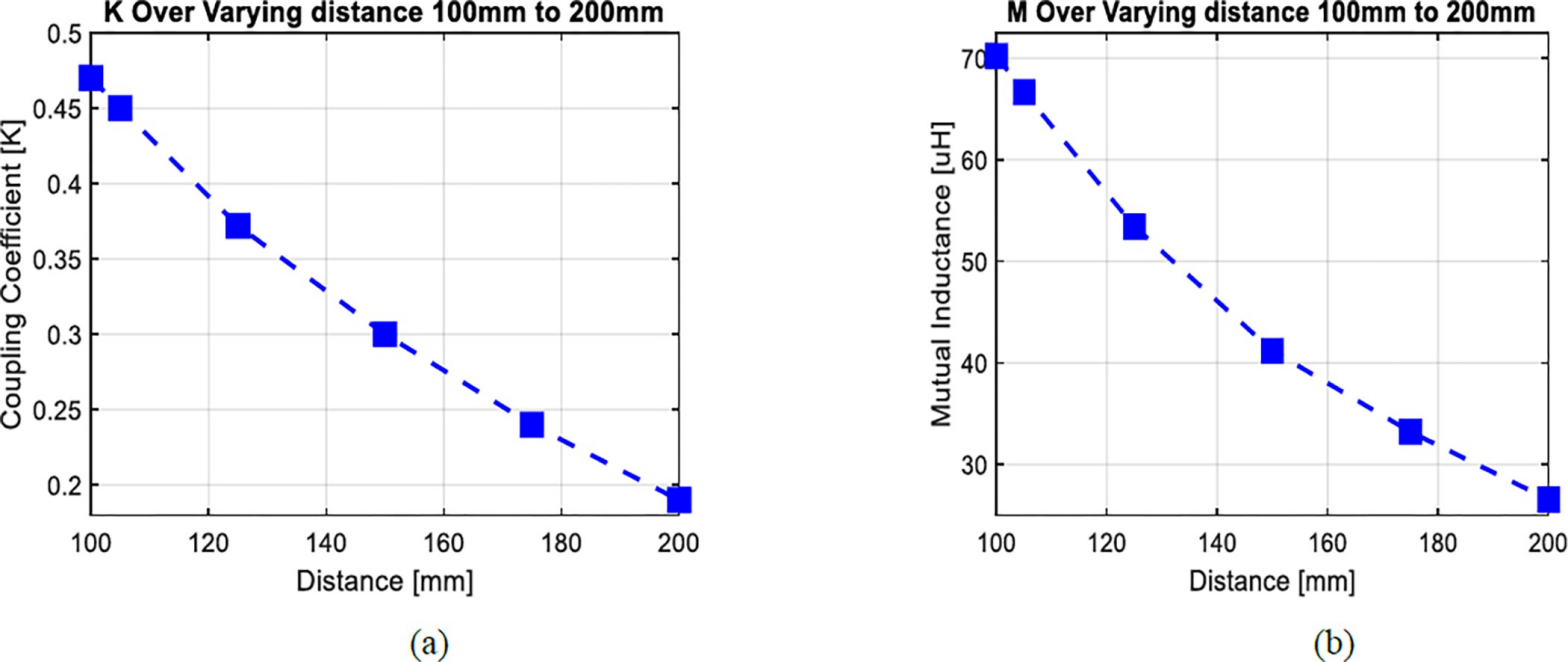
(a) The effect of vertical misalignment with ferrite bars (d = 100mm to 200mm) on coupling coefficient and (b) mutual inductance.

**Fig 11:**
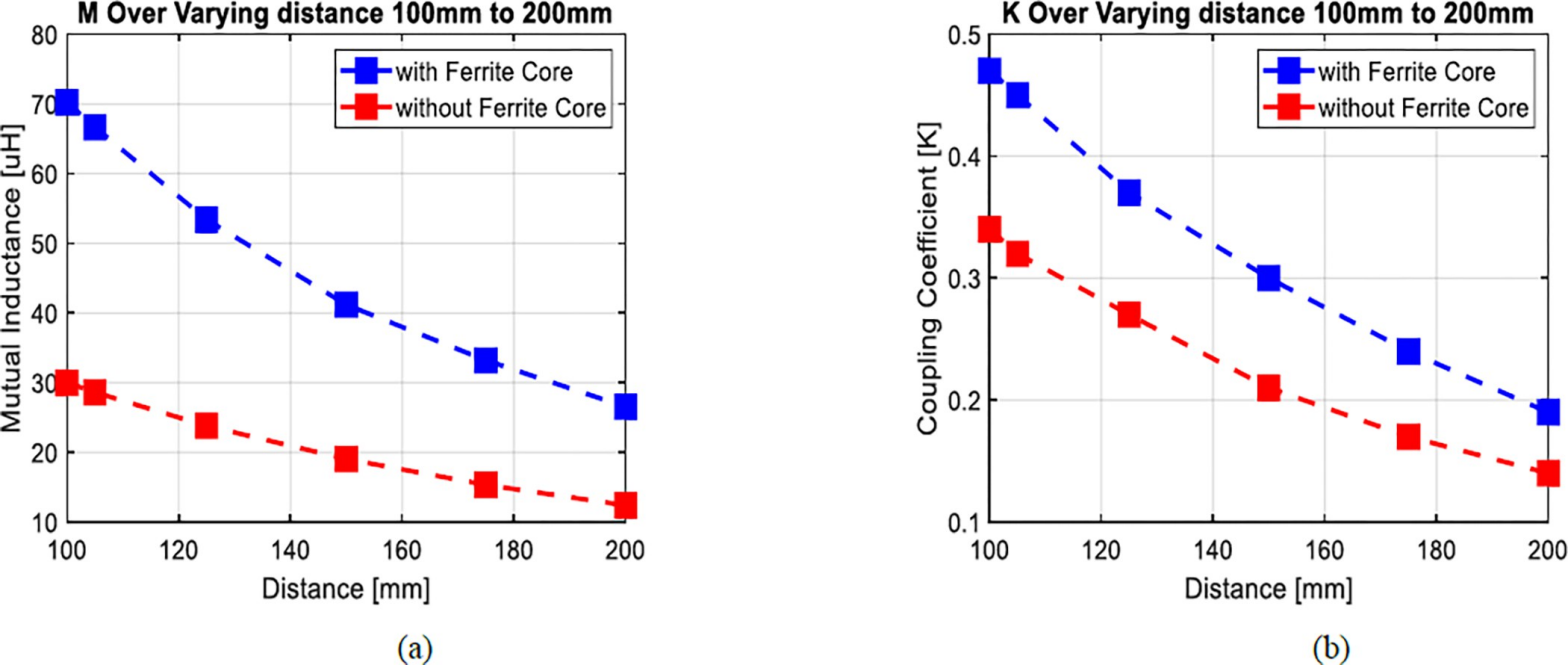
(a) The effect of vertical misalignment with ferrite bars (d = 100mm to 200mm) on coupling coefficient (b) mutual inductance.

**Fig 12:**
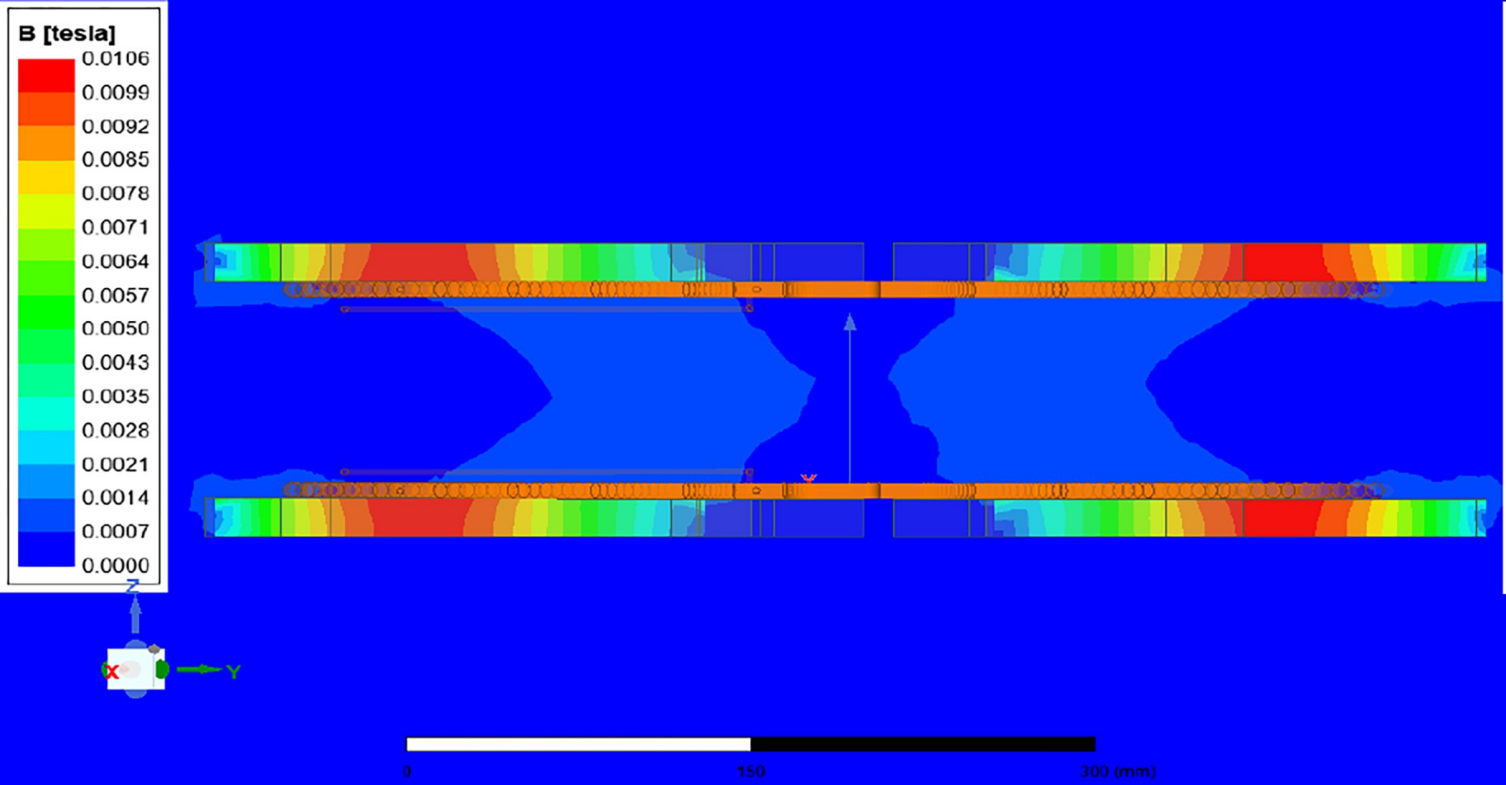
Magnetic field line distribution with ferrite at a distance of 105 mm.

Figs 10 and 11 reveal a clear correlation: an increase in the separation distance between coils leads to a simultaneous decrease in both the coupling coefficient and mutual inductance. However, the incorporation of ferrite material positively impacts the coupling coefficient, indicating enhanced energy transfer efficiency. Consequently, configurations with ferrite material demonstrate superior energy transmission compared to those without it. In Fig 12, the introduction of high magnetic permeability ferrite significantly influences magnetic field distribution, concentrating field lines and improving efficient transmission. These characteristic positions ferrite as a shielding material, contributing to enhanced magnetic field trapping.

## 4. The influence of system efficiency due to compensation in (SS)

**Fig 13:**
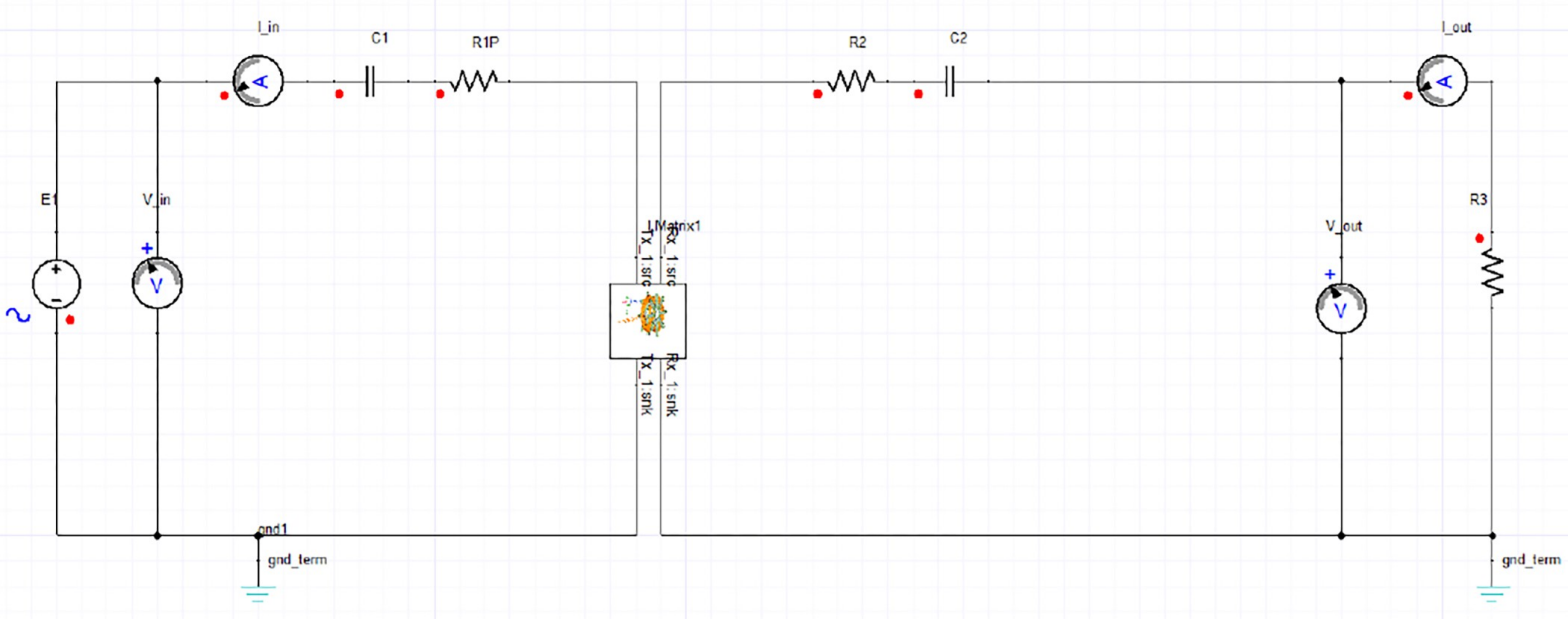
The electrical circuit with S-S compensation using ansys simpler.

The simulated circular coil with a Ferrite core at 85 KHz, as shown in Fig 13 and Table 3, was meticulously designed in Ansys Maxwell. The coil’s dynamic inductance specifics were then incorporated into Simplorer as inductance parameters (referred to as L parameters). The transmitting end featured a series connection of the resonance capacitor with the inductor to optimize current flow and maximize power delivery, while the receiving end also utilized a series connection of the capacitor and inductor to generate a significant voltage drop across the load resistor, thereby optimizing the system for power reception.

**Table 3:** Parameter values To transfer 3.6 KW.

Parameters	Value
Voltage	300 V
Frequency	85 KHz
R1	48.6 mΩ
R2	48.6 mΩ
C1	24 nF
C2	24 nF
R_load_	50 Ω

**Fig 14:**
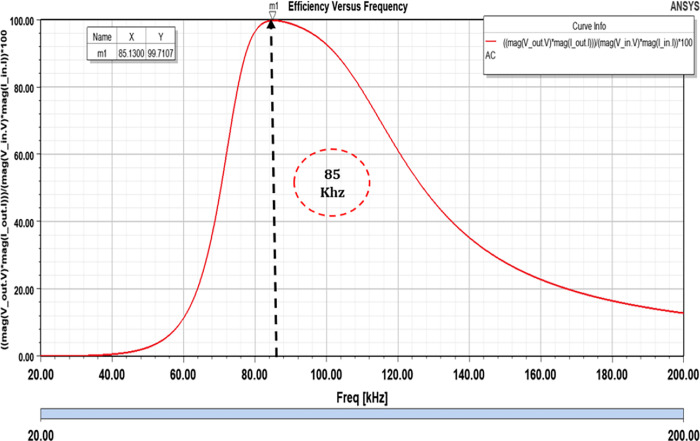
Efficiency versus frequency.

**Fig 15:**
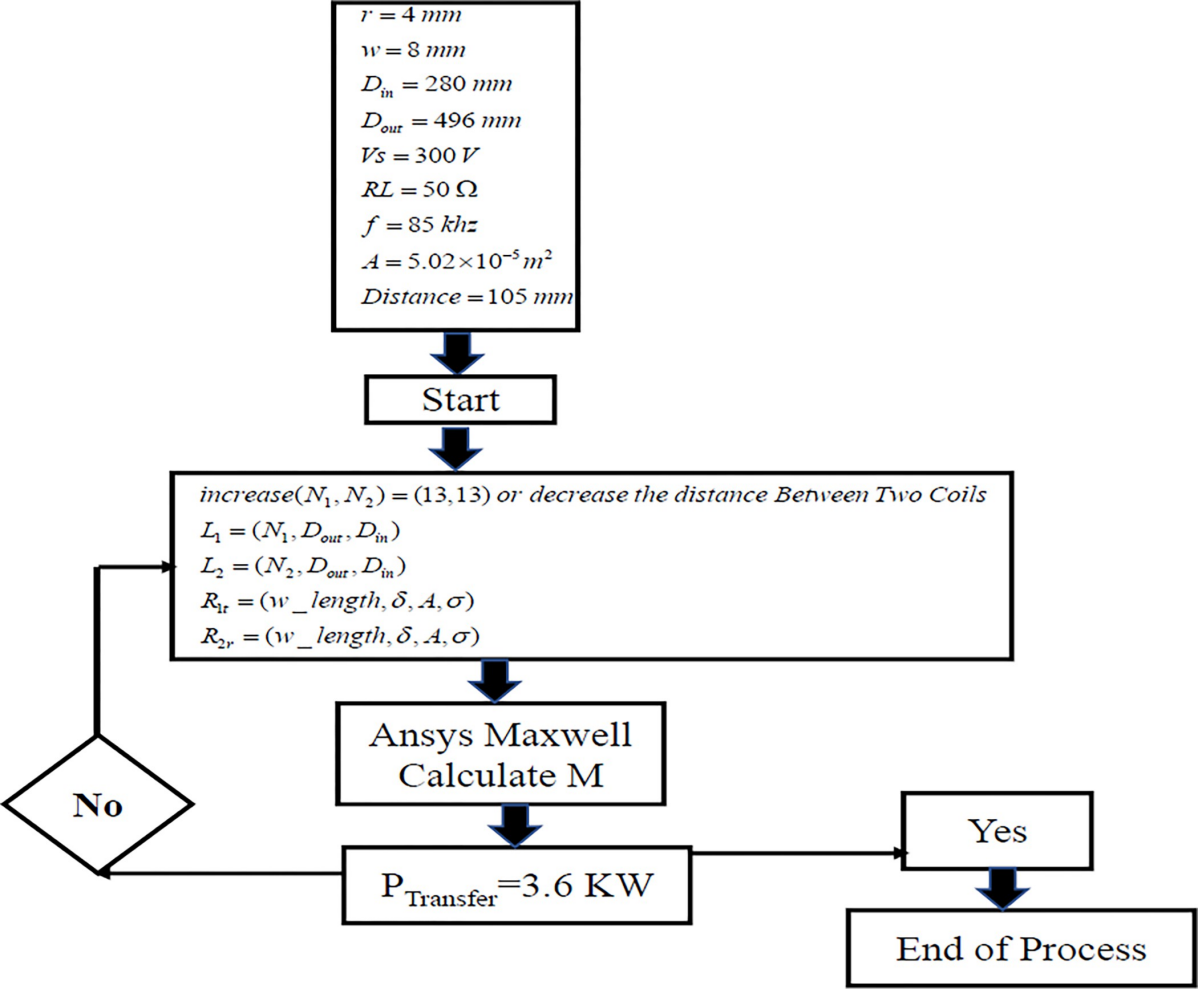
Diagram of the optimization process.

The simulations encompass an AC Analysis configuration aimed at assessing the system’s efficiency. This assessment involves a frequency span ranging from 20 kHz to 200 kHz. The outcomes of these simulations demonstrate that the designated dimensions effectively allow the system to achieve remarkable efficiency levels; reaching up to 99.02% for a power output of 3.6 kW we can also calculate it by MATLAB as shown in Figs 14 and 15.

## 5. Conclusions

This paper delves into the realm of magnetic resonant wireless systems, specifically focusing on S-S compensation. However, a notable hurdle persists in pinpointing the ideal parameters for the charging pad to maximize system efficiency. In addition, when the magnetic circuit is in free space, the magnetic field lines expand beyond the region between the two coils, necessitating the implementation of performance shielding. To address this issue, the study introduces the use of a ferrite which has a higher permeability that is placed on top of the coils. This positioning focuses on the flux lines within the energy transmission zone, leading to a 30% enhancement in the coupling efficiency and an increase in the transferred energy level. The study also presents a novel approach involving a circular spiral coil for series-series compensation, able to send 3.6 kilowatts of energy at a frequency of 85 kHz. The S-S topology emerges as the most favorable option due to its robustness against load and coupling fluctuations, ensuring peak charging performance. The simulation findings demonstrate that the system can attain an impressive efficiency of approximately 99%. In the context of these studies, it is possible to validate and experimentally test the results using various materials and shapes.
