# Correction: Enhancing electric vehicle charging performance through series-series topology resonance-coupled wireless power transfer

**DOI:** 10.1371/journal.pone.0309545

**Published:** 2024-08-22

**Authors:** Nadir Benalia, Idriss Benlaloui, Kouider Laroussi, Ahmad Elkhateb, Daniel Eutyche Mbadjoun Wapet, Ammar M. Hassan, Mohamed Metwally Mahmoud

There is an error in affiliation 2 for author Idriss Benlaloui. The correct affiliation 2 is: Department of Electrical Engineering, LSPIE Laboratory, University of Batna 2, Batna, Algeria.
