# Supplementary material for: Enhancing electric vehicle charging performance through series-series topology resonance-coupled wireless power transfer
Source: PLoS One. 2024 Mar 21;19(3):e0300550. doi: 10.1371/journal.pone.0300550 (PMC10956748; doi:10.1371/journal.pone.0300550)
Supplement: Attachment — Submitted filename: Authors Responses to Reviewers Plos one Round 1.docx [file pone.0300550.s001.docx]

**To,**

**The Editor-in-Chief and Reviewers**

**PLOS ONE**

Subject: Submission of revised manuscript on “Enhancing Electric Vehicle Charging Performance through Series-Series Topology Resonance-Coupled Wireless Power Transfer”

**Ref: # PONE-D-23-39863**

Dear Editor and Reviewers,

The authors would like to thank the editor and the respected reviewers for their precious time and invaluable and constructive comments on the work entitled **“*Enhancing Electric Vehicle Charging Performance through Series-Series Topology Resonance-Coupled Wireless Power Transfer”*.** Indeed, we are pleased that our paper has found a positive echo in your journal and it needs major revision. We have carefully taken into account all constructive comments and recommendation addressed by all the respected reviewers.

The authors have also gone through the manuscript and checked some typographical/grammatical errors in the manuscript. The manuscript has been also improved according to this issue.

The detailed responses to the reviewers’ comments are placed at the end of this letter. The revised portions of the manuscript have been marked in yellow.

We hope that the revised manuscript has been improved to reach the required satisfaction for publication in your esteemed journal. Weare eagerly waiting for your kind feedback and decision.

Please feel free to contact us if there are any further requirement to be carried out.

Thank you again for your kind patience and attention.

Sincerely Yours,

**Daniel Eutyche Mbadjoun Wapet**

Reviewer #2:

**Reviewer 2**

The responses and corrections concerning the comments and remarks of **reviewer 2** have been addressed carefully in the revised version of the paper.

First of wireless transfer using different converter is a nice concept to explore. Following are the observations;

**Comment 1:** Authors are claiming 3.6 kW power transfer, so to establish this concept what is the rating of the converter has chosen to fit in with wireless transfer for EVs?
**Authors’ response to comment 1:** Achieving a power transfer of 3.6 kW necessitates a converter with a rating of 5 kW. In an academic research context, the implementation could involve employing SiC switches for the inverter. One viable option is the C3M0040120D Silicon Carbide N-channel Power MOSFET. Such academic endeavors would contribute to the advancement of knowledge in wireless power transfer systems for EVs.

**Comment 2:** Table 1 please modify the inductance symbol.

**Authors’ response to comment 2:** Thank you once more for this precise comment. We agree with the respected reviewer about this comment. Accordingly, we have corrected this. We invite kindly the respected reviewer to check this update within the revised version of the paper.

**Comment 3:** The manuscript format should be as per journal's format.

**Authors’ response to comment 3:** We would like to thank the respected reviewer for this meticulous comment. We invite kindly the respected reviewer to check this update within the revised version of the paper

**Comment4:** Please format fig. 8 in one page.
**Authors’ response to comment 4:** We would like to thank the respected reviewer for this meticulous comment. We invite kindly the respected reviewer to check this update in page: 10 lines **…-….** in the revised manuscript.

Reviewer #1: 1. You ignored emphasizing the valid reason behind using the grid voltage's positive sequence voltages in the estimation of the unit templates and the possible impacts of distortions of the unbalanced and distorted grid voltages.

2. The exact role of the cascaded PI controller in the EV battery charging process and the mode the reference current is established for the current controller has not been stated.

3. You failed to explain the purposes of hysteresis current controller and the process which supports the generation of gating pulses for the VSC.

4. The different steps involved in the process of modifying the frequency of the AC voltages to achieve synchronization with the grid voltages were left out of the paper.

5. The function and responsibilities of the bidirectional dc-dc converter in regulating the dc link voltage were missing.

6. There is a failure to explore the control pulses for the VSC generated and what role the digital controller plays in implementing the control algorithm across different modes of operation.

7. The concept behind the PWM generator in the charging process and how it facilitates the control of the bidirectional converter has yet to be expounded.

8. What are the key advantages and applications of bidirectional power electronic switches (PES) in modern power systems, and how do they contribute to enhancing energy efficiency and grid flexibility?

9. I would recommend elucidating the key considerations, challenges, and technologies involved in transitioning power systems or devices from grid-connected mode to stand-alone mode, particularly in the context of ensuring reliability, resilience, and efficient energy management.

10. The main components and communication protocols involved in enabling bidirectional power flow between electric vehicles and the grid need to be described. At the same time, how does V2G contribute to the integration of renewable energy sources and grid stability?

11. The cascaded PI controller is used to charge an EV battery, but how these controllers are effective in charging the EV batteries needs to be described.

12.How do insulated gate bipolar junction transistors (IGBTs) function, and what are their key advantages and applications in power electronics, particularly in the context of high-power applications and energy-efficient systems?

All the reviewer comments are not related to our paper. We think it was submitted by error.

Authors hope that the learned Reviewer must be convinced with all the explanations and approve the paper for publication.
