# Supplementary material for: Enhancing electric vehicle charging performance through series-series topology resonance-coupled wireless power transfer
Source: PLoS One. 2024 Mar 21;19(3):e0300550. doi: 10.1371/journal.pone.0300550 (PMC10956748; doi:10.1371/journal.pone.0300550)
Supplement: Peer review history

# Peer review: Decision Letter 0

**DOI:** 10.1371/journal.pone.0300550.r001

**Authors:** Kalisadhan Mukherjee

4 Feb 2024

PONE-D-23-39863Enhancing Electric Vehicle Charging Performance through Series-Series Topology Resonance-Coupled Wireless Power TransferPLOS ONE

Dear Dr. Mbadjoun Wapet,

Thank you for submitting your manuscript to PLOS ONE. After careful consideration, we feel that it has merit but does not fully meet PLOS ONE’s publication criteria as it currently stands. Therefore, we invite you to submit a revised version of the manuscript that addresses the points raised during the review process.

Please submit your revised manuscript by Mar 20 2024 11:59PM. If you will need more time than this to complete your revisions, please reply to this message or contact the journal office at plosone@plos.org. When you're ready to submit your revision, log on to https://www.editorialmanager.com/pone/ and select the 'Submissions Needing Revision' folder to locate your manuscript file.

Please include the following items when submitting your revised manuscript:

- A rebuttal letter that responds to each point raised by the academic editor and reviewer(s). You should upload this letter as a separate file labeled 'Response to Reviewers'.
- A marked-up copy of your manuscript that highlights changes made to the original version. You should upload this as a separate file labeled 'Revised Manuscript with Track Changes'.
- An unmarked version of your revised paper without tracked changes. You should upload this as a separate file labeled 'Manuscript'.

If you would like to make changes to your financial disclosure, please include your updated statement in your cover letter. Guidelines for resubmitting your figure files are available below the reviewer comments at the end of this letter.

If applicable, we recommend that you deposit your laboratory protocols in protocols.io to enhance the reproducibility of your results. Protocols.io assigns your protocol its own identifier (DOI) so that it can be cited independently in the future. For instructions see: https://journals.plos.org/plosone/s/submission-guidelines#loc-laboratory-protocols. Additionally, PLOS ONE offers an option for publishing peer-reviewed Lab Protocol articles, which describe protocols hosted on protocols.io. Read more information on sharing protocols at https://plos.org/protocols?utm_medium=editorial-email&utm_source=authorletters&utm_campaign=protocols.

We look forward to receiving your revised manuscript.

Kind regards,

Kalisadhan Mukherjee

Academic Editor

PLOS ONE

Journal Requirements:

When submitting your revision, we need you to address these additional requirements.

1. Please ensure that your manuscript meets PLOS ONE's style requirements, including those for file naming. The PLOS ONE style templates can be found at 

https://journals.plos.org/plosone/s/file?id=wjVg/PLOSOne_formatting_sample_main_body.pdf and 

https://journals.plos.org/plosone/s/file?id=ba62/PLOSOne_formatting_sample_title_authors_affiliations.pdf

2. Please note that PLOS ONE has specific guidelines on code sharing for submissions in which author-generated code underpins the findings in the manuscript. In these cases, all author-generated code must be made available without restrictions upon publication of the work. Please review our guidelines at https://journals.plos.org/plosone/s/materials-and-software-sharing#loc-sharing-code and ensure that your code is shared in a way that follows best practice and facilitates reproducibility and reuse.

Additional Editor Comments:

Please carefully address the comments raised by the reviewers.

[Note: HTML markup is below. Please do not edit.]

Reviewers' comments:

Reviewer's Responses to Questions

**Comments to the Author**

1. Is the manuscript technically sound, and do the data support the conclusions?

The manuscript must describe a technically sound piece of scientific research with data that supports the conclusions. Experiments must have been conducted rigorously, with appropriate controls, replication, and sample sizes. The conclusions must be drawn appropriately based on the data presented. 

Reviewer #1: Partly

Reviewer #2: Yes

**********

2. Has the statistical analysis been performed appropriately and rigorously? 

Reviewer #1: No

Reviewer #2: Yes

**********

3. Have the authors made all data underlying the findings in their manuscript fully available?

The PLOS Data policy requires authors to make all data underlying the findings described in their manuscript fully available without restriction, with rare exception (please refer to the Data Availability Statement in the manuscript PDF file). The data should be provided as part of the manuscript or its supporting information, or deposited to a public repository. For example, in addition to summary statistics, the data points behind means, medians and variance measures should be available. If there are restrictions on publicly sharing data—e.g. participant privacy or use of data from a third party—those must be specified.

Reviewer #1: Yes

Reviewer #2: Yes

**********

4. Is the manuscript presented in an intelligible fashion and written in standard English?

PLOS ONE does not copyedit accepted manuscripts, so the language in submitted articles must be clear, correct, and unambiguous. Any typographical or grammatical errors should be corrected at revision, so please note any specific errors here.

Reviewer #1: No

Reviewer #2: Yes

**********

5. Review Comments to the Author

Please use the space provided to explain your answers to the questions above. You may also include additional comments for the author, including concerns about dual publication, research ethics, or publication ethics. (Please upload your review as an attachment if it exceeds 20,000 characters)

Reviewer #1: 1. You ignored emphasizing the valid reason behind using the grid voltage's positive sequence voltages in the estimation of the unit templates and the possible impacts of distortions of the unbalanced and distorted grid voltages.

2. The exact role of the cascaded PI controller in the EV battery charging process and the mode the reference current is established for the current controller has not been stated.

3. You failed to explain the purposes of hysteresis current controller and the process which supports the generation of gating pulses for the VSC.

4. The different steps involved in the process of modifying the frequency of the AC voltages to achieve synchronization with the grid voltages were left out of the paper.

5. The function and responsibilities of the bidirectional dc-dc converter in regulating the dc link voltage were missing.

6. There is a failure to explore the control pulses for the VSC generated and what role the digital controller plays in implementing the control algorithm across different modes of operation.

7. The concept behind the PWM generator in the charging process and how it facilitates the control of the bidirectional converter has yet to be expounded.

8. What are the key advantages and applications of bidirectional power electronic switches (PES) in modern power systems, and how do they contribute to enhancing energy efficiency and grid flexibility?

9. I would recommend elucidating the key considerations, challenges, and technologies involved in transitioning power systems or devices from grid-connected mode to stand-alone mode, particularly in the context of ensuring reliability, resilience, and efficient energy management.

10. The main components and communication protocols involved in enabling bidirectional power flow between electric vehicles and the grid need to be described. At the same time, how does V2G contribute to the integration of renewable energy sources and grid stability?

11. The cascaded PI controller is used to charge an EV battery, but how these controllers are effective in charging the EV batteries needs to be described.

12.How do insulated gate bipolar junction transistors (IGBTs) function, and what are their key advantages and applications in power electronics, particularly in the context of high-power applications and energy-efficient systems?

Reviewer #2: First of wireless transfer using different converter is a nice concept to explore. Following are the observations;

1. Authors are claiming 3.6kW power transfer, so to establish this concept what is the rating of the converter has chosen to fit in with wireless transfer for EVs?

2.Table 1 please modify the inductance symbol.

3. The manuscript format should be as per journal's format.

4. Please format fig. 8 in one page.

**********

6. PLOS authors have the option to publish the peer review history of their article (what does this mean?). If published, this will include your full peer review and any attached files.

If you choose “no”, your identity will remain anonymous but your review may still be made public.

**Do you want your identity to be public for this peer review?** For information about this choice, including consent withdrawal, please see our Privacy Policy.

Reviewer #1: No

Reviewer #2: No

**********

[NOTE: If reviewer comments were submitted as an attachment file, they will be attached to this email and accessible via the submission site. Please log into your account, locate the manuscript record, and check for the action link "View Attachments". If this link does not appear, there are no attachment files.]

While revising your submission, please upload your figure files to the Preflight Analysis and Conversion Engine (PACE) digital diagnostic tool, https://pacev2.apexcovantage.com/. PACE helps ensure that figures meet PLOS requirements. To use PACE, you must first register as a user. Registration is free. Then, login and navigate to the UPLOAD tab, where you will find detailed instructions on how to use the tool. If you encounter any issues or have any questions when using PACE, please email PLOS at figures@plos.org. Please note that Supporting Information files do not need this step.

# Author comment: Author response to Decision Letter 0

**DOI:** 10.1371/journal.pone.0300550.r002

11 Feb 2024

To,

The Editor-in-Chief and Reviewers

PLOS ONE 

Subject: Submission of revised manuscript on “Enhancing Electric Vehicle Charging Performance through Series-Series Topology Resonance-Coupled Wireless Power Transfer” 

Ref: # PONE-D-23-39863

Dear Editor and Reviewers,

The authors would like to thank the editor and the respected reviewers for their precious time and invaluable and constructive comments on the work entitled “Enhancing Electric Vehicle Charging Performance through Series-Series Topology Resonance-Coupled Wireless Power Transfer”. Indeed, we are pleased that our paper has found a positive echo in your journal and it needs major revision. We have carefully taken into account all constructive comments and recommendation addressed by all the respected reviewers. 

The authors have also gone through the manuscript and checked some typographical/grammatical errors in the manuscript. The manuscript has been also improved according to this issue.

The detailed responses to the reviewers’ comments are placed at the end of this letter. The revised portions of the manuscript have been marked in yellow. 

We hope that the revised manuscript has been improved to reach the required satisfaction for publication in your esteemed journal. Weare eagerly waiting for your kind feedback and decision.

Please feel free to contact us if there are any further requirement to be carried out.

Thank you again for your kind patience and attention.

Sincerely Yours,

Daniel Eutyche Mbadjoun Wapet 

Reviewer #2: 

Reviewer 2

The responses and corrections concerning the comments and remarks of reviewer 2 have been addressed carefully in the revised version of the paper.

First of wireless transfer using different converter is a nice concept to explore. Following are the observations;

Comment 1: Authors are claiming 3.6 kW power transfer, so to establish this concept what is the rating of the converter has chosen to fit in with wireless transfer for EVs?

Authors’ response to comment 1: Achieving a power transfer of 3.6 kW necessitates a converter with a rating of 5 kW. In an academic research context, the implementation could involve employing SiC switches for the inverter. One viable option is the C3M0040120D Silicon Carbide N-channel Power MOSFET. Such academic endeavors would contribute to the advancement of knowledge in wireless power transfer systems for EVs.

Comment 2: Table 1 please modify the inductance symbol.

Authors’ response to comment 2: Thank you once more for this precise comment. We agree with the respected reviewer about this comment. Accordingly, we have corrected this. We invite kindly the respected reviewer to check this update within the revised version of the paper.

Comment 3: The manuscript format should be as per journal's format.

Authors’ response to comment 3: We would like to thank the respected reviewer for this meticulous comment. We invite kindly the respected reviewer to check this update within the revised version of the paper

Comment4: Please format fig. 8 in one page.

Authors’ response to comment 4: We would like to thank the respected reviewer for this meticulous comment. We invite kindly the respected reviewer to check this update in page: 10 lines …-…. in the revised manuscript.

Attachment Submitted filename: Authors Responses to Reviewers Plos one Round 1.docx

# Peer review: Decision Letter 1

**DOI:** 10.1371/journal.pone.0300550.r003

**Authors:** Kalisadhan Mukherjee

29 Feb 2024

Enhancing Electric Vehicle Charging Performance through Series-Series Topology Resonance-Coupled Wireless Power Transfer

PONE-D-23-39863R1

Dear Dr. Mbadjoun Wapet,

We’re pleased to inform you that your manuscript has been judged scientifically suitable for publication and will be formally accepted for publication once it meets all outstanding technical requirements.

Within one week, you’ll receive an e-mail detailing the required amendments. When these have been addressed, you’ll receive a formal acceptance letter and your manuscript will be scheduled for publication.

An invoice for payment will follow shortly after the formal acceptance. To ensure an efficient process, please log into Editorial Manager at http://www.editorialmanager.com/pone/, click the 'Update My Information' link at the top of the page, and double check that your user information is up-to-date. If you have any billing related questions, please contact our Author Billing department directly at authorbilling@plos.org.

If your institution or institutions have a press office, please notify them about your upcoming paper to help maximize its impact. If they’ll be preparing press materials, please inform our press team as soon as possible -- no later than 48 hours after receiving the formal acceptance. Your manuscript will remain under strict press embargo until 2 pm Eastern Time on the date of publication. For more information, please contact onepress@plos.org.

Kind regards,

Kalisadhan Mukherjee

Academic Editor

PLOS ONE

Additional Editor Comments (optional):

Reviewers have recommended acceptance of the manuscript. The manuscript is thus accepted for publication.

Reviewers' comments:

Reviewer's Responses to Questions

**Comments to the Author**

1. If the authors have adequately addressed your comments raised in a previous round of review and you feel that this manuscript is now acceptable for publication, you may indicate that here to bypass the “Comments to the Author” section, enter your conflict of interest statement in the “Confidential to Editor” section, and submit your "Accept" recommendation.

Reviewer #1: All comments have been addressed

Reviewer #2: All comments have been addressed

**********

2. Is the manuscript technically sound, and do the data support the conclusions?

The manuscript must describe a technically sound piece of scientific research with data that supports the conclusions. Experiments must have been conducted rigorously, with appropriate controls, replication, and sample sizes. The conclusions must be drawn appropriately based on the data presented. 

Reviewer #1: Yes

Reviewer #2: Yes

**********

3. Has the statistical analysis been performed appropriately and rigorously? 

Reviewer #1: Yes

Reviewer #2: Yes

**********

4. Have the authors made all data underlying the findings in their manuscript fully available?

The PLOS Data policy requires authors to make all data underlying the findings described in their manuscript fully available without restriction, with rare exception (please refer to the Data Availability Statement in the manuscript PDF file). The data should be provided as part of the manuscript or its supporting information, or deposited to a public repository. For example, in addition to summary statistics, the data points behind means, medians and variance measures should be available. If there are restrictions on publicly sharing data—e.g. participant privacy or use of data from a third party—those must be specified.

Reviewer #1: Yes

Reviewer #2: Yes

**********

5. Is the manuscript presented in an intelligible fashion and written in standard English?

PLOS ONE does not copyedit accepted manuscripts, so the language in submitted articles must be clear, correct, and unambiguous. Any typographical or grammatical errors should be corrected at revision, so please note any specific errors here.

Reviewer #1: Yes

Reviewer #2: Yes

**********

6. Review Comments to the Author

Please use the space provided to explain your answers to the questions above. You may also include additional comments for the author, including concerns about dual publication, research ethics, or publication ethics. (Please upload your review as an attachment if it exceeds 20,000 characters)

Reviewer #1: Accepted for publication in this esteemed journal

all questions have been answered

figures are now correct

grammar is correct

Reviewer #2: All the comments are addressed. Just want to highlight in fig 4(b) Dout where 't' is cut slightly. How ever during proof reading with the permission of the Editor it can be corrected. Best wishes.

**********

7. PLOS authors have the option to publish the peer review history of their article (what does this mean?). If published, this will include your full peer review and any attached files.

If you choose “no”, your identity will remain anonymous but your review may still be made public.

**Do you want your identity to be public for this peer review?** For information about this choice, including consent withdrawal, please see our Privacy Policy.

Reviewer #1: No

Reviewer #2: No

**********

# Editor report: Acceptance letter

**DOI:** 10.1371/journal.pone.0300550.r004

**Authors:** Kalisadhan Mukherjee

7 Mar 2024

PONE-D-23-39863R1 

PLOS ONE

Dear Dr. Mbadjoun Wapet, 

I'm pleased to inform you that your manuscript has been deemed suitable for publication in PLOS ONE. Congratulations! Your manuscript is now being handed over to our production team.

At this stage, our production department will prepare your paper for publication. This includes ensuring the following:

* All references, tables, and figures are properly cited

* All relevant supporting information is included in the manuscript submission,

* There are no issues that prevent the paper from being properly typeset

If revisions are needed, the production department will contact you directly to resolve them. If no revisions are needed, you will receive an email when the publication date has been set. At this time, we do not offer pre-publication proofs to authors during production of the accepted work. Please keep in mind that we are working through a large volume of accepted articles, so please give us a few weeks to review your paper and let you know the next and final steps. 

Lastly, if your institution or institutions have a press office, please let them know about your upcoming paper now to help maximize its impact. If they'll be preparing press materials, please inform our press team within the next 48 hours. Your manuscript will remain under strict press embargo until 2 pm Eastern Time on the date of publication. For more information, please contact onepress@plos.org.

If we can help with anything else, please email us at customercare@plos.org.

Thank you for submitting your work to PLOS ONE and supporting open access. 

Kind regards, 

PLOS ONE Editorial Office Staff

on behalf of

Dr. Kalisadhan Mukherjee 

Academic Editor

PLOS ONE
